# On the use of ZIP codes and ZIP code tabulation areas (ZCTAs) for the spatial analysis of epidemiological data

**DOI:** 10.1186/1476-072X-5-58

**Published:** 2006-12-13

**Authors:** Tony H Grubesic, Timothy C Matisziw

**Affiliations:** 1 Department of Geography, Indiana University, Bloomington, IN 47405-7100, USA; 2 Center for Urban and Regional Analysis, The Ohio State University, Columbus, OH 43210-1361, USA

## Abstract

**Background:**

While the use of spatially referenced data for the analysis of epidemiological data is growing, issues associated with selecting the appropriate geographic unit of analysis are also emerging. A particularly problematic unit is the ZIP code. Lacking standardization and highly dynamic in structure, the use of ZIP codes and ZIP code tabulation areas (ZCTA) for the spatial analysis of disease present a unique challenge to researchers. Problems associated with these units for detecting spatial patterns of disease are explored.

**Results:**

A brief review of ZIP codes and their spatial representation is conducted. Though frequently represented as polygons to facilitate analysis, ZIP codes are actually defined at a narrower spatial resolution reflecting the street addresses they serve. This research shows that their generalization as continuous regions is an imposed structure that can have serious implications in the interpretation of research results. ZIP codes areas and Census defined ZCTAs, two commonly used polygonal representations of ZIP code address ranges, are examined in an effort to identify the spatial statistical sensitivities that emerge given differences in how these representations are defined. Here, comparative analysis focuses on the detection of patterns of prostate cancer in New York State. Of particular interest for studies utilizing local, spatial statistical tests, is that differences in the topological structures of ZIP code areas and ZCTAs give rise to different spatial patterns of disease. These differences are related to the different methodologies used in the generalization of ZIP code information. Given the difficulty associated with generating ZIP code boundaries, both ZIP code areas and ZCTAs contain numerous representational errors which can have a significant impact on spatial analysis. While the use of ZIP code polygons for spatial analysis is relatively straightforward, ZCTA representations contain additional topological features (e.g. lakes and rivers) and contain fragmented polygons that can hinder spatial analysis.

**Conclusion:**

Caution must be exercised when using spatially referenced data, particularly that which is attributed to ZIP codes and ZCTAs, for epidemiological analysis. Researchers should be cognizant of representational errors associated with both geographies and their resulting spatial mismatch, especially when comparing the results obtained using different topological representations. While ZCTAs can be problematic, topological corrections are easily implemented in a geographic information system to remedy erroneous aggregation effects.

## Background

As the production and consumption of spatial data continues to increase, the subsequent use and abuse of spatially referenced data is also on the rise. Jacquez [1] provides a timely review of the key issues, outlining a number of limitations to working with spatial and temporal data. For example, one of the major issues confronting analysts is spatiotemporal mismatch. Broadly defined, this occurs when data collected in both space and time do not coincide. For example, Jacquez [1] highlights a recent study of lung cancer on Long Island that used cancer data collected at the ZIP+4 level reported for 1994–97 [2]. Cancer incidence was then compared to air toxics data from the Environmental Protection Agency for 1996. In this particular instance, the mismatch is both spatial and temporal.

A second concern highlighted by Jacquez [1] and others [3-5] is the issue of granularity in epidemiological data. In sum, granularity deals with the spatial and temporal resolution of data. Because human health applications must adhere to patient privacy protocols, individual level data is frequently aggregated to larger spatial units for analysis. For instance, rather than utilizing geocoded household data corresponding to individual patients, these records are aggregated to the ZIP code level for analysis. This process prevents unwanted disclosure or reconstruction of patient identity [1]. However, it also reduces the ability for analysts to compare data across spatial units. For example, if one set of data is aggregated to census tracts and another set to ZIP codes, issues relating to the modifiable areal unit problem emerge [6].

A third major issue of interest is more technical in nature, that of polygons, topology and computational geometry. As noted by Jacquez [1], many spatial statistical techniques are predicated on the accurate representation of areal units (polygons), points and lines. If there are problems with areal units, such as self intersection, the resulting statistical analyses can be interlaced with errors.

As with most technical issues, epidemiologists, geographers and other analysts are aware of the limitations and caveats of working with spatial data. For example, in a study of cerebrovascular disease in New York State, Han et al. [7] note:

"[t]here may be some bias related to spatial mismatch, since we have used ZIP-code level hospitalization data and ZCTA-level population and income data in our analysis.... Unfortunately, we could not find any empirical study that validates this issue of spatial mismatch."

Of particular interest in the previous statement is the issue of bias and spatial mismatch between ZIP code areas and ZIP code tabulation areas (ZCTA). In fact, the problems of spatiotemporal mismatches between these two units have largely gone unnoticed. While Kreiger et al. [8] provide a brief overview regarding many of the technical differences between ZIP codes and ZCTAs, a full treatise of the differences, particularly how these differences may bias empirical analysis, is not available.

The purpose of this study is to 1) reexamine the use and misuse of ZIP codes and ZCTAs for epidemiological analysis, 2) provide enough technical detail on the construction of ZIP code and ZCTA boundaries, and their associated characteristics, to supply analysts with a more complete picture of their utility for spatial analysis, 3) provide an empirically based analysis of the spatial and statistical mismatch between ZIP code areas and ZCTAs, highlighting their relative weaknesses, and 4) develop a methodological approach for rectifying the problems inherent to ZCTA topologies, so that more direct comparisons between ZCTA and ZIP code-based analysis may be performed.

## Results and discussion

### Issues of spatial misrepresentation and mismatch

In the context of longitudinal spatial analyses, the ability to match spatial units through time is important. Fortunately, the hierarchically nested spatial units provided by the Census Bureau (e.g. blocks, block groups, tracts, counties, etc.) simplify this task. In most cases, changes to the spatial structure of Census tracts and even block groups, can be tracked between the decennial surveys. As a result, accurate longitudinal analyses are much easier to perform. However, for temporally and spatially dynamic areal units that are not hierarchically nested, the problems of spatiotemporal mismatch are significant. Not surprisingly, the ZIP code and its spatial characteristics are of concern. Exceedingly popular for epidemiological analysis, the ZIP code has become a de-facto spatial unit for the study of disease distribution and etiology [9-13].

Zone Improvement Plan codes, or ZIP codes as they are commonly known, originated as a way of classifying street segments, address ranges and delivery points to expedite the delivery of mail. Given that ZIP codes can be associated with most places of human habitation in the United States, they present researchers with an alternative means of collecting, visualizing, and analyzing spatial information. However, given their use in directing the distribution of mail, ZIP codes are not attributed to space in general, but rather to roads, post offices, and other facilities within the U.S. postal system. For instance, if an area does not have a recognized delivery point or address range, no ZIP code is assigned. Geographically, the best examples of this are in desolate and uninhabited places such as the Sonora Desert in Arizona, the Mojave Desert in California and the Klamath Mountains in Oregon. Simply put, if no residential areas or business establishments exist, there is no need to deliver mail or assign a five digit ZIP code. The process for making ZIP codes accessible for spatial analysis, has involved their generalization into polygonal units representing the spatial extent of ZIP code delivery areas (referred to here as ZIP code areas). In large part, the tiling of the United States with ZIP code areas has been accomplished by various private data vendors. More recently, the U.S. Census Bureau has produced its own ZIP code topology for area based representations – ZIP Code Tabulation Areas (ZCTAs).

The use of ZIP codes for applications other than postal delivery can present many challenges and there are several major issues worth summarizing. First, the United States Postal Service (USPS) makes updates to its ZIP codes regularly [14], providing this information in the biweekly Postal Bulletin. However, for analysts unfamiliar with a particular area, understanding the magnitude and nature of these changes is a challenge. For example, it is not uncommon for postal delivery routes to be realigned or for ZIP codes to be split. More importantly, ZIP codes can be discontinued, added or expanded between months/years. Thus, where longitudinal studies are concerned, even the slightest modification in ZIP codes and their associated coverage can create a spatiotemporal discontinuity [8]. Many private data vendors update ZIP code area databases quarterly. However, even this relatively short time-lag between updates can be problematic for areas where significant changes were made, particularly for syndromic surveillance or infectious outbreaks. Further, if analysts fail to make use of available updates, problems can also emerge. Another difficulty associated with ZIP code areas is the significant variation in geographic extent [8,10]. Grubesic [15] notes that the average size of a ZIP code area in Wyoming is (1,430 square kilometers), while the average size of a ZIP code area in New Jersey is 12.8 km^2^. The USPS does attempt to optimize the size or population allocation of ZIP codes given that the sole purpose of the ZIP code is to expedite the delivery of mail. As a result, ZIP codes can range in size from a single building to a delivery zone spanning hundreds of square miles and crossing several political jurisdictions [16].

As mentioned earlier, ZCTAs were developed as spatial units by the U.S. Census Bureau for the 2000 decennial census. In fact, ZCTAs were specifically designed to "meet requests by data users for statistical data by ZIP Code area" [17]. Given the Census Bureau's motivations, Krieger et al. [8] note that there are significant differences in the technical definitions of ZIP codes areas and ZCTAs. Table 1 highlights the technical details of ZCTAs. First, ZCTAs can be *discontiguous*. By definition, spatial contiguity refers to the ability to travel from any point in a polygon to any other internal point without leaving it. Where two or more polygons are considered, spatial contiguity is the property of sharing a common boundary or vertex [18]. The lack of spatial contiguity can have a dramatic impact on spatial statistical analysis, particularly if ZCTAs with a common identifier are split into different non-adjacent polygons. Second, ZCTAs are compiled based on census block topology. In the generation of a ZCTA, each underlying block is assigned one, and only one, ZCTA code – regardless of its location. Therefore, it is possible for blocks to straddle more than one ZCTA or ZIP code. This can be problematic when aggregating population data to both units.

**Table 1:** A Summary of Census ZCTA Characteristics

1.	ZCTAs are linked to Census blocks and every tabulation block has a single ZCTA code
2.	ZCTAs cover all tabulation blocks in the United States and Puerto Rico
3.	ZCTAs may consist of two or more *discontiguous *areas
4.	A ZCTA code represents a five digit ZIP code where possible
5.	In large undeveloped areas where there are no master address file (MAF) addresses with five-digit ZIP codes, the ZCTA code assigned is based on the three-digit ZIP code (e.g. XX for tracts of undeveloped land and HH for water features)

To provide some perspective on the extent of these problems, consider the following. Table 2 highlights the numerical differences between unedited ZIP code and ZCTA geographic base files (GBF) available for New York State. In addition to there being 851 additional entries/polygons in the ZCTA file, the average size of these polygons is significantly smaller (51.90 km^2 ^v. 70.26 km^2^) than those found in the ZIP code GBF. While the numerical characteristics of these files are certainly different, these statistics only hint at to the severity of spatial mismatch present between these two geographies.

**Table 2:** Numerical Differences between ZCTA and ZIP Code Geographic Base Files in New York State

	**ZCTA (2000)**	**ZIP Code (GDT 2000)**
	
Number of Polygons	2,450	1,599
Number of Unique Records	1,676	1,599
Average Size	51.90 km^2^	70.26 km^2^
Minimum Size	0.003 km^2^	0.006 km^2^
Maximum Size	1,054 km^2^	1,217 km^2^
Standard Deviation in Size	80.34 km^2^	102.71 km^2^

**Figure 1:**
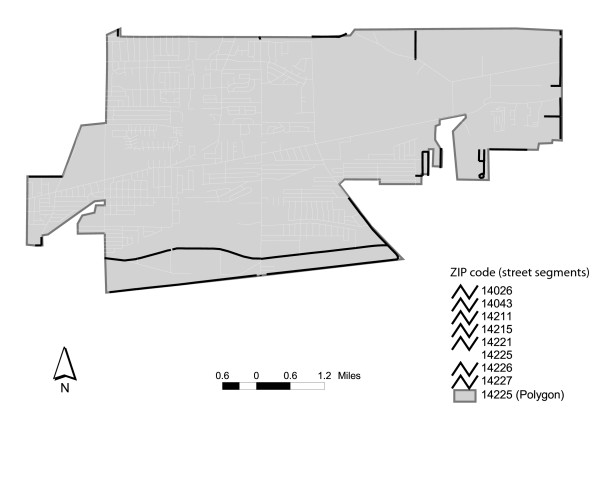
Non-native zip code segments within 14225 (Buffalo, NY).

As discussed earlier, ZIP code information is often used to generate polygonal representations of ZIP code delivery areas. During this conversion process, the vast majority of the spatial mismatch problems begin to arise. In large part, this can be attributed to attempts to generalize linear features (i.e. street segments) into zones for representational convenience [15]. For instance, Figure 1 illustrates ZIP code 14225 in Buffalo, New York. In this example, the ZIP code boundary is clearly demarcated as a discrete unit by polygonal boundaries [19]. However, because ZIP codes are, in fact, associated with linear features, the actual boundaries of 14225 are not so clear-cut. As displayed in Figure 1, there are a total of seven other streets in the 14225 polygon that actually belong to alternative ZIP codes. The implications for such spatial misrepresentations can be problematic, particularly if one considers the application of geocoded data for epidemiological analysis [20]. When individual records are geocoded to a street address, point-based representations of latitude and longitude coordinates are assigned to a street centerline, and then placed at an appropriate offset distance to represent the location of a household or business [21,22]. However, if the actual location of the street segment and its associated centerline deviates from its "native" ZIP code polygon, both uncertainty and error *can *be introduced to the analysis, even if the geocode is a perfect match. For example, a geocoded point might be assigned to the correct ZIP code, based on the underlying network data, but the ZIP code area or ZCTA covering its actual location could be different. In other words, the network data and the ZIP polygons are not in correspondence. Therefore, although the data was accurately aggregated to the appropriate ZIP code, its spatial representation will not be accurately accounted for in the analysis. Similarly, if patients' ZIP codes are collected and attributed to polygons based on an obsolete ZIP code topology, error is also introduced. Further, even when public health agencies avoid more traditional geocoding routines (i.e. point-based representation of latitude and longitude coordinates) problems may emerge. For example, situations exist where geocoding based on the street network can fail. In these cases, analysts may to attribute ZIP code information based on visual inspection, possible resulting in a misclassification. While one or two of these errors might not make a significant difference to a local study, the accumulation of error for statewide or national-level analyses can be significant.

**Figure 2:**
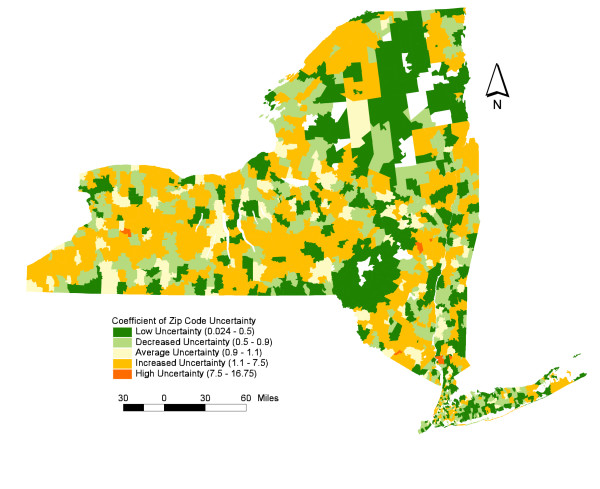
Coefficient of ZIP Code Uncertainty: Shown is a map of GDT ZIP code areas and the level of uncertainty associated with each.

In an effort to diagnose the local level of uncertainty associated with the problem of non-native street segments within ZIP code polygons, consider Figure 2. Displayed are the results of a calculation developed for this paper called the Coefficient of ZIP Code Uncertainty, or C*ZU*_*i*_. *CZU*_*i *_measures the local concentration of non-native street segments within a ZIP code area relative to the number of non-native segments for all ZIP codes in New York State. As a diagnostic, the resulting index values provide a baseline measure of spatial uncertainty and potential representational error associated with each ZIP code. The interpretation of *CZU*_*i *_is as follows:

*CZU*_*i *_<1 = decreased level of uncertainty

*CZU*_*i *_= 1 = average level of uncertainty

*CZU*_*i *_>1 = increased level of uncertainty

Figure 2 suggests that while many of the GDT ZIP codes in New York State include fewer than expected numbers of non-native street segments, many others display an increased level of uncertainty. Clearly, this suggests the presence of a relatively substantial gap between the ZIP codes assigned to linear features and their location relative to interpolated ZIP code areas. Interestingly, much of this uncertainty can be attributed to the process of ZIP code polygon interpolation, which is outlined in the next section.

### ZIP code polygon interpolation

The process for developing ZIP code area polygons is relatively laborious. As mentioned previously, these areal units are not developed and distributed by the USPS [15]. Rather, private data vendors, such as GDT/TeleAtlas [19] and Caliper [23] generate these boundaries. Boundaries are created by using several important pieces of information. First, data vendors leverage mail-stop (i.e. residential and business addresses) information from the USPS and their associated street segments. Second, other non-street features are also analyzed, including water bodies, parks, and large tracts of undeveloped land. Third, ZIP+4 state directories are used to differentiate delivery zones and the corresponding boundaries for areas that might not have a clear-cut group of street segments. Finally, technicians make telephone inquiries to area post offices in an effort to determine predominant ZIP codes [24]. Once all of this information is collected, ZIP code polygons are manually digitized. This process, particularly the use of manual digitizing routines, can lead to polygon generalization and a "smoother" geographic boundary file.

The process for developing ZCTAs by the U.S. Census Bureau is much different. As highlighted in Table 1, ZCTAs have some relatively distinct features that ZIP codes do not. Many of these features relate to the characteristics of the Census blocks on which they are based. There is no standard spatial extent of Census blocks. Some blocks are relatively small (i.e. those located in a city), while others are large and irregular, covering many square miles. Utilizing Census block boundaries, USPS ZIP code data and the 2000 Master Address File (MAF)[25], the Census Bureau calculated the numbers of addresses associated with each ZIP code represented in each tabulation block and then assigned the ZCTA that represented the most frequently occurring ZIP code with preference given to residential addresses. If no ZIP code data were available, ZCTA codes were assigned from an adjoining block. Finally, it is important to remember that since the size of Census blocks vary widely over space, zone delineation is guided more by the Census geographies than by the distribution of ZIP coded addresses.

**Figure 3:**
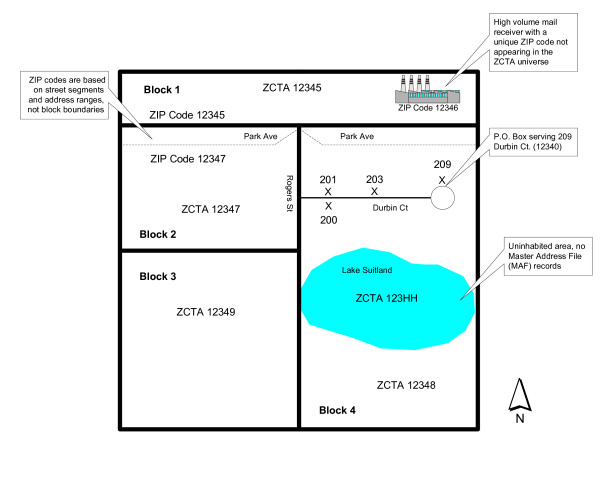
Example ZCTA Neighborhood.

Figure 3 displays an example neighborhood that graphically highlights a few of these quirks. For instance, the United States Postal Service assigns a ZIP code of 12345 to both sides of Park Ave, but assigns a ZIP code of 12347 to segments south of Park Ave, including Rogers St. While this appears to be an oddity, the USPS often utilizes rear property lines for assigning ZIP codes [17]. Therefore, the resulting ZIP code polygon that straddles both sides of Park Ave. is not surprising. However, this geographic quirk is not characteristic of ZCTAs, because blocks are assigned one, and only one, ZCTA code. Therefore, because Park Ave. is the dividing segment between two blocks, the entire south side of Park Ave. inherits an erroneous ZCTA code of 12347, instead of its correct ZIP code of 12345. A second interesting example is illustrated by the factory located in ZCTA 12345, which is assigned a ZIP code of 12346. In many instances, USPS customers that receive an extraordinarily high volume of mail are assigned their own ZIP code. This might be a large corporate campus or other institution. Because these locations are treated as delivery *points *by the USPS, they are systematically excluded by the Census Bureau and do not appear in the ZCTA boundary file. This is understandable since these delivery points do not have any spatial boundaries nor are they associated with any census related demographic or socioeconomic information. Also, the inability to precisely locate structures and a lack of available block boundaries for many of these locales influences the decision to exclude many of these features. Finally, the Census Bureau assigns three digit ZIP codes (e.g. 123 HH) to areas associated with water features and where no entries exist within the Master Address File (MAF). However, because Census blocks were developed *before *ZCTAs, the resulting ZCTA boundaries had to conform to tabulation block boundaries. As a result, any attempts to assign water bodies, such as a river, to a ZCTA would result in a polygon with a tail-like feature. In an effort to avoid these problems, the Census Bureau designated these areas with the alphanumeric code rather than a five-digit ZCTA. In other cases (not displayed in Figure 3), this might include a 123XX code. The XX codes are assigned to large tracts of land where no mailing addresses are located and no ZIP codes are maintained by the USPS. The decision to assign a three-digit wildcard ZCTA code (e.g. HH or XX) to some areas in the United States is a complex and speculative one [17]. Given that ZCTA geographies incorporate these additional landscape features, problems often arise in assessing ZCTA contiguity.

**Figure 4:**
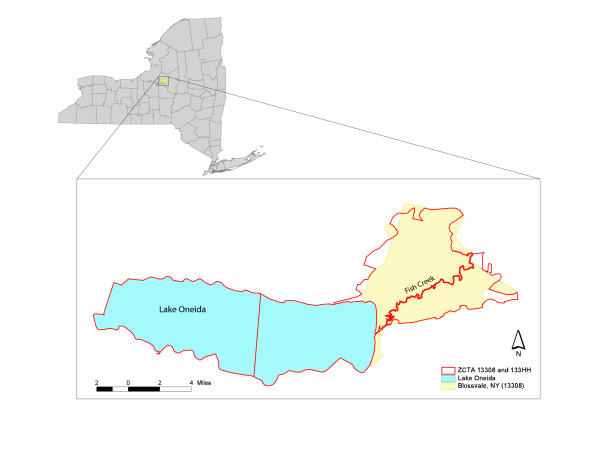
Topological Disruption: Shown is ZIP code area 13308 and its companion 13308 ZCTA for Blossvale, NY. Also displayed is Lake Oneida and the 133 HH code for the corresponding water bodies in the ZCTA geographic base file.

For example, to illustrate the topological problems that water features create in the ZCTA geographic base file, observe Figure 4. Illustrated is Blossvale, NY (13308), a small community near Syracuse, located north of Interstate 90 and about 2 miles northeast of Lake Oneida. The 13308 ZIP code (as merged by the New York State Department of Health), also includes the communities of Sylvan Beach, North Bay, Verona Beach and McConnellsville.

The standard GDT (2000) ZIP code boundaries for Blossvale are highlighted in yellow. The ZCTA boundaries for the same ZIP code and the neighboring Lake Oneida are displayed in red. There are several critical points worth addressing here. First, the 13308 ZCTA and GDT ZIP code area representations are not in complete spatial correspondence, given that there are a number of slight deviations between these two areal units. Clearly, this represents a spatial mismatch. Second, notice that a small water feature, Fish Creek, cuts the 13308 ZCTA in half. When one examines the raw geographic base files for ZCTAs, 13308 actually appears *twice*. That is, there are two separate and distinct entries in the geographic base file for the 13308 ZCTA. Thus, if the ZCTA remains uncorrected, data assigned to the ZCTA will be represented twice. Additionally, if an adjacency matrix is constructed, as is often necessary in spatial statistical analysis, the 13308 ZCTAs are *not *treated as neighbors because they are split by the 130 HH water feature polygon. Therefore, inclusion of these polygons can muddle spatial relationships between ZCTAs that have socioeconomic, demographic and epidemiologic data associated with them. Clearly, any lack of adjustment to the ZCTA geographic base file incorporates these types of errors into the subsequent analysis.

Given this background in ZIP code area interpolation and ZCTA development, there are several questions remaining to be answered. First, how do these potential spatial inconsistencies manifest in the real-world? Second, what kind of impact would these problems have on spatial-statistical analysis? Third, how does one correct these problems to ensure consistency and accuracy in an analysis?

### Mitigating topological anomalies in the ZCTA geographic base file

To illustrate some of the issues associated with use of ZIP code areas and ZCTAs in spatial analysis, both topologies for New York State were obtained for analysis. In order to compare ZIP code areas with ZCTAs in New York, several important steps must be undertaken to mitigate the topological anomalies between these two geographic base files. Based on year 2000 ZIP code data from GDT, New York is covered by 1,599 ZIP code areas. Conversely, 2,450 Census ZCTAs cover the state (Table 2). In part, this high number of ZCTAs is a product of the 398 water features found in the state that fragment the ZCTAs. To bring these two geographies into greater accord, several steps must be taken to adjust the ZCTA file for the presence of these features [15]:

1. In order to rectify the topological anomalies in the ZCTA file, one must remove all ZCTAs with HH codes. This eliminates all water features in the file. While the features are still visible, they are no longer entities in the geographic base file. It is not as critical to remove features with XX codes, because these actually do represent land masses with no formal addresses in the system, rarely splitting a ZCTA into multiple features like a river or creek might (See Figure 4).

2. All five-digit ZCTA entries that consist of multiple polygons (e.g. split by a water feature) must be dissolved on a common attribute ID. In virtually every case, this can be the ZCTA code. The dissolve process merges polygons into single features, removing double or triple entries in the geographic base file and ignoring any splits in polygon continuity that may have been created by water features.

3. Cancer incident cases, population, or whatever variables of interest are being analyzed, must be reaggregated back to the topologically rectified ZCTA geographic base file for analysis. This effectively removes the aggregation errors (e.g. double counting) from the original file.

4. Finally, if one is conducting a spatial statistical analysis that relies on neighborhood information, the adjacency matrix must be recalculated using the rectified ZCTA file. Again, because the water features are removed, and ZCTA polygons are now dissolved on a common attribute, the newly calculated adjacency matrix will represent a more realistic and accurate snapshot of spatial relationships between polygons.

After correcting for the water polygons, the ZCTA and ZIP code area boundary files are in nearly complete correspondence. For the analysis that follows, ZIP code based prostate incidence data was obtained from the New York State Department of Health (NYSDOH) [26]. As discussed in the methodology section, data for some ZIP code areas were aggregated in this particular dataset. In an attempt to accurately represent this data, both the New York ZIP code area and ZCTA geographies used in this analysis were subject to similar aggregation of areas where necessary. Given this aggregation, the GDT ZIP code areas, subsequently modified to meet confidentiality requirements by the NYSDOH, numbered 1,384 while the topologically adjusted ZCTA file now includes 1,389 areas – yielding a difference of only 5 polygons. This small difference can be attributed to five partitions of land with no five-digit ZIP codes – areas maintained by the Census Bureau in the ZCTA file (i.e. XX codes).

### Statistical mismatch

**Figure 5:**
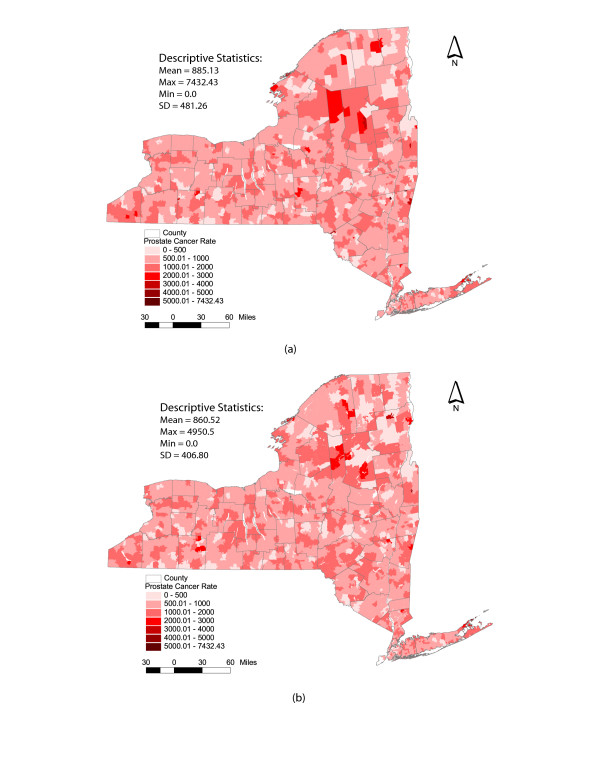
Prostate Cancer Rates in New York State – 1999 – 2003. (a) GDT ZIP code boundaries; (b) Census 2000 ZCTA boundaries.

Figure 5 displays incidence of prostate cancer in New York State for 1999–2003 which was collected from the New York State Cancer Registry [26]. Specifically, Figure 5a illustrates the prostate cancer rates using the ZIP code polygons based on modified GDT data. In contrast, Figure 5b illustrates prostate cancer rates using ZCTA polygons from the year 2000 distributed from the U.S. Census Bureau. Cartographically, there is little discernable difference between these two maps. Given this distribution of rates, a formal epidemiological analysis might seek an approach that facilitates the identification of high-risk ZIP codes or groups of ZIP codes for intervention. Such analysis might also benefit from the identification of low-risk ZIP codes or groups of ZIP codes for additional exploration. For example, Han et al. [7] utilized ZCTAs and cluster analysis to explore the geographical variation of cerebrovascular disease in New York State, while Moonan et al., [27] used ZCTAs and basic cartographic analysis to examine areas of tuberculosis transmission and incidence.

**Figure 6:**
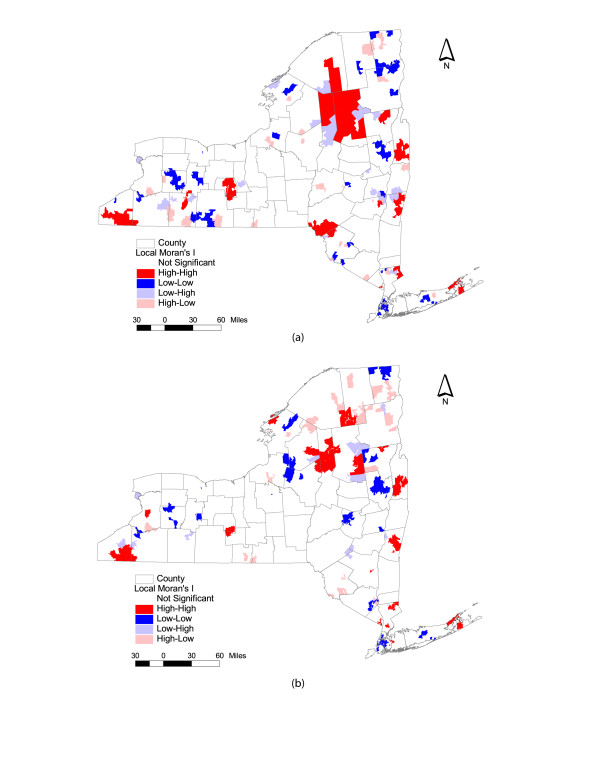
Clustering of Prostate Cancer Rates in New York State: 1999 – 2003. Shown are maps that display cluster memberships derived by a local indicator of spatial association (Moran's I). (a) GDT ZIP code boundaries; (b) Census 2000 ZCTA boundaries.

For the purposes of this study, our goal does not include a formal epidemiological analysis of prostate cancer, per se. We are primarily interested in identifying the potential spatial and statistical mismatches between results obtained through the use of ZIP codes area and ZCTA geographies. Interestingly, Figure 5 indicates relatively substantial differences in prostate cancer rates when comparing the descriptive statistics between ZIP code areas and ZCTAs. As noted in the introductory section, topological issues associated with these areal units are critical when conducting spatial statistical analyses. In an effort to illustrate the problem of spatial mismatch and the impact of topology, consider Figure 6. Figure 6a illustrates statistically derived prostate cancer clusters for New York State, generated using a local indicator of spatial association (Moran's I) [28,29], based here upon a first order queen's contiguity. Specifically, the areas represented in Figure 6 correspond to one of five classifications generated through the test of local spatial association. For example, areas denoted in the darker red are indicative of ZIP code areas of high prostate cancer rates which are surrounded by other high rate ZIP code areas. Conversely, ZIP codes denoted in the darker blue color are indicative of low rate areas surrounded by other low rate areas. The remaining classifications are high-low, low-high and not significant (*p *<= 0.05). It is important to note that Figure 6a utilizes the GDT ZIP code areas while Figure 6b utilizes ZCTAs. When comparing these two figures, there are some remarkable differences in the statistical results. Even the simplest visual inspection suggests that these patterns of spatial association between ZIP code areas and ZCTA data do not match, even though the underlying data on prostate cancer incidence is identical. For example, Figure 6a displays a relatively large area of high-high ZIP codes in the Adirondacks and several low-low clusters in portions of Western New York and Long Island. This is not corroborated by the pattern generated using ZCTAs as units of analysis. Statistically, the differences are also relatively obvious. For instance, there are 108 ZIP code areas classified as low-low clusters in New York. Conversely, only 96 ZCTAs are classified as low-low.

**Figure 7:**
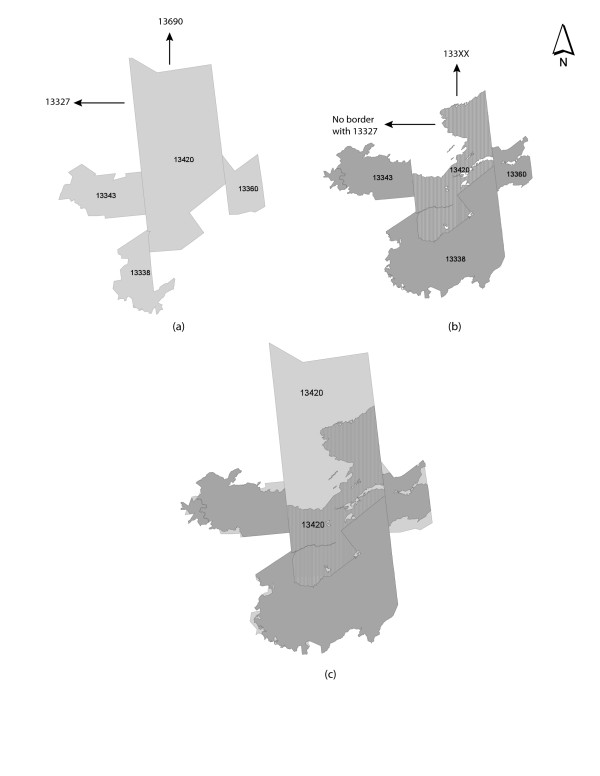
Spatial Mismatch Between ZIP Code Areas and ZCTAs. (a) GDT ZIP code area boundaries; (b) Census 2000 ZCTA boundaries; (c) Shown is a composite map of 13420, 13360 13338 and 13343 in New York State.

There are four major reasons these differences in patterns emerge. First, although all of the ZIP code areas and ZCTAs share identical identifier codes (e.g. 12065), this does not guarantee that they share the same geographic boundary or extent. For example, Figure 7 illustrates a composite map of four ZIP code areas and ZCTAs in Upstate New York. In this case, there is a clear difference in spatial extent and bounding between the two geographic base files. As a result, when a spatial weights matrix is constructed, the local neighborhoods for each of these ZIP codes will be different. Further, once a statistical test is constructed for examining local spatial association, the derived results will also be different (see Figure 6). A second factor relates to the inclusion of XX coded ZCTAs in the spatial adjacency matrix. While it is possible to remove these polygons, the resulting map does not convey the true geography of New York State. Moreover, because these polygons do represent a landmass, it is important to include them to assure the continuity of the spatial weights matrix. A third problem relates to how other spatial data can be associated with these units the ZIP code areas and ZCTAs. For example, in this study, Census block population data are used to calculate prostate cancer rates. Specifically, male population for each block was aggregated to each ZIP code area and ZCTA, ensuring that each block was only counted once. Clearly, if the ZIP code area and ZCTA polygons are different in spatial extent, the results of this aggregation process will differ. As Figures 5 and 6 suggest, these differences can substantially impact the resulting analysis. Finally, many of the more obvious spatial mismatches in New York are in sparsely populated areas such as the Adirondack Mountains. In part, this can be attributed to the sensitivity of the local Moran's I test to low population counts. In these instances, cluster results can fluctuate dramatically based on small differences in observed cases [30]. That said, there are still numerous cases of spatial mismatch in heavily populated areas, particularly Long Island.

In summary, ZIP code areas and ZCTAs are not directly comparable units of observation. In addition to displaying significant differences in size and extent, there is a major disconnect in the way these units are generated. These differences stem from the fact that ZIP codes are based on address ranges, developed for mail delivery and their representation as polygons does not accurately portray all of the linear features in a ZIP code. Given the methods by which these areal units are generated, there are many instances where ZIP ranges are misclassified by ZIP code areas and ZCTAs. Our research also suggests that ZCTAs present some challenges with which analysts must address, particularly in their spatial representation. As noted previously, Census blocks are used for building ZCTA boundaries. In addition to the errors introduced by representing linear features with polygons, each block is assigned a single ZCTA code. While this is good for looking at census data, if there is overlap or underlap between ZIP code segments, the ZCTA zoning scheme is unable to accurately portray these differences. Further, the incorporation of water features and uninhabited areas into the ZCTA geographic base file can also complicate spatial analysis.

In conclusion, the problem of spatiotemporal mismatch is significant for ZIP codes and ZCTAs. Caution must be used when attempting to compare statistical results across both time and space when these units are used. More importantly, analysts must also weigh the cost/time benefits of rectifying ZCTA topology for conducting epidemiological analysis. While this certainly involves more work and GIS processing time, the benefits of these modifications are significant.

## Methods

### Data

Observed values of prostate cancer incidence were retrieved from the New York State Cancer Registry. ZIP code boundaries were created by Geographic Data Technology for the year 2000 and subsequently modified by the NYSDOH [26]. These modifications include the following:

1. Some adjacent ZIP codes were combined due to confidentiality requirements because an insufficient numbers of cases of prostate cancer were reported.

2. A subset of residential point ZIP codes with no defined delivery area and ZIPs too small to be included in the GDT file were also combined with adjacent ZIP code areas.

3. NYSDOH also eliminated uninhabited islands from the ZIP code area file.

ZCTA boundaries were delineated by the U.S. Census Bureau for the year 2000. The street network used for calculating *CZU*_*i *_were based on TIGER 2000 data [23].

### Modeling

The coefficient of ZIP code uncertainty is calculated as follows:

CZUi=xi/yi∑inxi/∑inyi     (Equation 1)
 MathType@MTEF@5@5@+=feaafiart1ev1aaatCvAUfKttLearuWrP9MDH5MBPbIqV92AaeXatLxBI9gBaebbnrfifHhDYfgasaacH8akY=wiFfYdH8Gipec8Eeeu0xXdbba9frFj0=OqFfea0dXdd9vqai=hGuQ8kuc9pgc9s8qqaq=dirpe0xb9q8qiLsFr0=vr0=vr0dc8meaabaqaciaacaGaaeqabaqabeGadaaakeaacqWGdbWqcqWGAbGwcqWGvbqvdaWgaaWcbaGaemyAaKgabeaakiabg2da9maalaaabaWaaSGbaeaacqWG4baEdaWgaaWcbaGaemyAaKgabeaaaOqaaiabdMha5naaBaaaleaacqWGPbqAaeqaaaaaaOqaamaalyaabaWaaabCaeaacqWG4baEdaWgaaWcbaGaemyAaKgabeaaaeaacqWGPbqAaeaacqWGUbGBa0GaeyyeIuoaaOqaamaaqahabaGaemyEaK3aaSbaaSqaaiabdMgaPbqabaaabaGaemyAaKgabaGaemOBa4ganiabggHiLdaaaaaakiaaxMaacaWLjaWaaeWaaeaacqqGfbqrcqqGXbqCcqqG1bqDcqqGHbqycqqG0baDcqqGPbqAcqqGVbWBcqqGUbGBcqqGGaaicqaIXaqmaiaawIcacaGLPaaaaaa@5860@

Where

*x_i _*=the number of non-native ZIP code street segments in ZIP code *i*

*y_i _*= the number of street segments in ZIP code *i*

As mentioned previously, *CZU*_*i *_measures the local concentration of non-native street segments within a ZIP code area relative to the number of non-native segments for a larger spatial unit (e.g. a metropolitan area or a state). Segments with no ZIP codes were not included in this computation given that there is no way of telling whether or not they actually contained an address and which ZIP it was attributed to. It is also important to remember that *CZU*_*i *_says nothing about the length of these street segments. However, with a slight adjustment to both the numerator and denominator, the magnitude of uncertainty, as measured by the distance associated with each non-native street segment could be quantified.

ZIP code and ZCTA contiguity measurements were quantified through the use of a spatial weights matrix, ***W***. Elements of ***W ***are specified as:

wij=cij∑j=1ncij     (Equation 2)
 MathType@MTEF@5@5@+=feaafiart1ev1aaatCvAUfKttLearuWrP9MDH5MBPbIqV92AaeXatLxBI9gBaebbnrfifHhDYfgasaacH8akY=wiFfYdH8Gipec8Eeeu0xXdbba9frFj0=OqFfea0dXdd9vqai=hGuQ8kuc9pgc9s8qqaq=dirpe0xb9q8qiLsFr0=vr0=vr0dc8meaabaqaciaacaGaaeqabaqabeGadaaakeaacqWG3bWDdaWgaaWcbaGaemyAaKMaemOAaOgabeaakiabg2da9maalaaabaGaem4yam2aaSbaaSqaaiabdMgaPjabdQgaQbqabaaakeaadaaeWbqaaiabdogaJnaaBaaaleaacqWGPbqAcqWGQbGAaeqaaaqaaiabdQgaQjabg2da9iabigdaXaqaaiabd6gaUbqdcqGHris5aaaakiaaxMaacaWLjaWaaeWaaeaacqqGfbqrcqqGXbqCcqqG1bqDcqqGHbqycqqG0baDcqqGPbqAcqqGVbWBcqqGUbGBcqqGGaaicqaIYaGmaiaawIcacaGLPaaaaaa@50DA@

Where *c_ij _*= 1 if *i *and *j *share a common boundary or vertex; 0 otherwise. For the purposes of this study, first order properties include only those vertices and boundaries that are contiguous to the observation (ZIP code or ZCTA) in question (viz. a Queen's contiguity matrix). While there are alternatives to this spatial weight matrix (e.g. rook, or distance based), the selection of a queen's based measure provided an effective approach for highlighting the topological complexities of the ZCTA geographic base layer. A more robust contiguity matrix, using other spatial lags, or polygon boundary lengths would be appropriate for a formal analysis of cancer incidence and clustering.

The statistical analysis of local spatial association was conducted by using a local Moran's I test statistic. The local Moran's *I *[28] is defined as:

Ii=zi∑jwijzj     (Equation 3)
 MathType@MTEF@5@5@+=feaafiart1ev1aaatCvAUfKttLearuWrP9MDH5MBPbIqV92AaeXatLxBI9gBaebbnrfifHhDYfgasaacH8akY=wiFfYdH8Gipec8Eeeu0xXdbba9frFj0=OqFfea0dXdd9vqai=hGuQ8kuc9pgc9s8qqaq=dirpe0xb9q8qiLsFr0=vr0=vr0dc8meaabaqaciaacaGaaeqabaqabeGadaaakeaacqWGjbqsdaWgaaWcbaGaemyAaKgabeaakiabg2da9iabdQha6naaBaaaleaacqWGPbqAaeqaaOWaaabuaeaacqWG3bWDdaWgaaWcbaGaemyAaKMaemOAaOgabeaaaeaacqWGQbGAaeqaniabggHiLdGccqWG6bGEdaWgaaWcbaGaemOAaOgabeaakiaaxMaacaWLjaWaaeWaaeaacqqGfbqrcqqGXbqCcqqG1bqDcqqGHbqycqqG0baDcqqGPbqAcqqGVbWBcqqGUbGBcqqGGaaicqaIZaWmaiaawIcacaGLPaaaaaa@4DA2@

Where

*x*_*i *_and *x*_*j *_are observations for locations *i *and *j *(with mean *μ*)

*z*_*i *_= (*x*_*i *_- *μ*),

*z*_*j *_= (*x*_*j *_- *μ*), and

*w*_*ij *_= spatial weights matrix with values of 0 or 1.

## Authors' contributions

THG designed the study, conducted the analysis, drafted the manuscript and developed the coefficient of ZIP code uncertainty. TCM collaborated on the design of the analysis, manuscript revisions and coded several of the processes in TransCad and ArcGIS.
